# Prevention of LPS-Induced Microglia Activation, Cytokine Production and Sickness Behavior with TLR4 Receptor Interfering Peptides

**DOI:** 10.1371/journal.pone.0060388

**Published:** 2013-03-28

**Authors:** Dustin J. Hines, Hyun B. Choi, Rochelle M. Hines, Anthony G. Phillips, Brian A. MacVicar

**Affiliations:** Department of Psychiatry and Brain Research Centre, University of British Columbia, Vancouver, British Columbia, Canada; Boston University School of Medicine, United States of America

## Abstract

The innate immune receptor Toll-like 4 (TLR4) is the receptor activated by lipopolysaccharide (LPS), and TLR4-LPS interaction is well known to induce an innate immune response, triggering sickness behavior. Within the brain, TLR4 is highly expressed in brain microglia, and excessive inflammation resulting from activation of this pathway in the brain has been implicated in depressive disorders and neurodegenerative pathologies. We hypothesized that blocking LPS-induced activation of TLR4 would prevent downstream immune signaling in the brain and suppress the induction of sickness behavior. We used interfering peptides to block TLR4 activation and confirmed their efficacy in preventing second messenger activation and cytokine production normally induced by LPS treatment. Further, these peptides blocked morphological changes in microglia that are typically induced by LPS. We also demonstrated that intraperitoneal (i.p.) injection of Tat-TLR4 interfering peptides prevented LPS-induced sickness behavior, as assessed in home cage behavior and with the intracranial self-stimulation paradigm. These newly synthesised peptides inhibit TLR4 signaling thereby preventing changes in behavior and motivation caused by inflammatory stimuli. These peptides highlight the roll of TLR4 and microglia morphology changes in sickness behavior, and thus may be of therapeutic value in limiting the deleterious impact of excessive inflammation in specific CNS pathologies.

## Introduction

Small amounts of lipopolysaccharide (LPS) from invading bacteria are one of the first signals detected by the body upon infection, and detection of LPS primes the immune system to mount a defence. Following the onset of a typical infection, individuals display a coordinated set of behavioral conditions, known collectively as sickness behavior [1], [2], [3], that reflect a normal acute response to inflammation. The profound changes which constitute sickness behavior include loss of motivation for food and drink, diminished social interaction, fatigue, irritability, depression and cognitive impairment [3]. The expression of sickness behavior relies on motivational reorganization of priorities, which are dependent on the biological state of the animal and therefore can lead to diverse behavioral outcomes. Separate from this, sickness behavior also includes an element of altered motility, which is characteristic in sick animals. Under some circumstances, the initial inflammatory response can become uncontrolled and ultimately lead to other deleterious effects including prolonged inflammation and cytokine release which is known to contribute to CNS dysfunction, chronic depressive disorders and neurodegenerative processes [2], [4], [5]. Although the CNS actions of cytokines have been implicated in sickness behavior [6], [7], [8], the mechanisms in the brain that trigger this behavioral response are not well understood.

The Toll-like receptor 4 (TLR4) and its potent ligand LPS, represent one of the first and best characterized ligand and receptor combinations of the innate immune system [9], [10]. TLR4 receptors are expressed on microglia in the CNS and on cells of the immune system throughout the body [2], [11]. Systemic LPS acts on the CNS through several parallel pathways (reviewed in [11]) including: 1) activation of TLR4 on microglia in regions where the blood brain barrier (BBB) is permeable (e.g. area postrema and circumventricular organ [12]; 2) activation of perivascular cells and endothelial cells of blood vessels in the brain [13]; 3) stimulation of the afferent vagal nerves; and 4) transport across the BBB of cytokines generated by peripheral cells [14]. There is however, disagreement in the literature as to what extent each of these pathways contributes to the effects of the LPS driven inflammatory cascade.

Here we show that Tat-coupled interfering peptides block TLR4 signaling to second messengers and subsequent cytokine production normally induced by LPS, block morphological changes in microglia induced by LPS, and also prevent LPS-induced sickness behavior. We used multiple indices of sickness behavior including various measures of motor performance (open field and modified SHIRPA screen), as well as indices of motivation including titrated intracranial self stimulation (ICSS). Remarkably, these newly synthesized peptides prevent changes in behavior and motivation normally caused by inflammatory stimuli by inhibiting TLR4 signaling. These peptides highlight the roll of TLR4 and microglia in sickness behavior, and thus may be of therapeutic value in limiting the deleterious impact of excessive inflammation in specific CNS pathologies.

## Results

**Figure 1:**
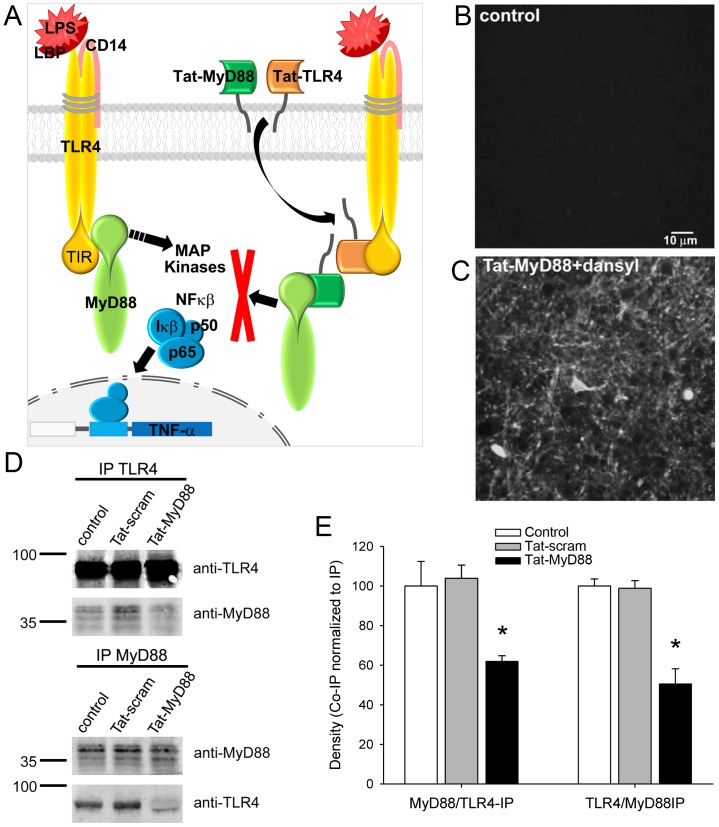
Schematic diagram showing the mechanism of action of the Tat-MyD88 and Tat-TLR4 peptides and their efficacy in preventing protein interactions *in vivo*. A. The peptides are directed against regions of the TLR4 receptor and MyD88 TIR domain, thereby interfering with the interaction of these two proteins. LPS treatment has been shown to increase TLR4 and MyD88 binding leading to the activation of MAP kinases and NFκβ modulation of TNF-α. Thus the peptides may be effective in blocking downstream signalling to MAP kinases and TNF-α. B,C. 2-photon images of hippocampal tissue following intraperitoneal (i.p.) injection in the mouse reveals that dansylated Tat peptide can be observed in brain cells. D When i.p. injected, Tat-MyD88 but not Tat-scram reduced co-immunoprecipitation of TLR4 and MyD88 from brain tissue. E Densitometry quantification of co-immunoprecipitated protein normalized to immunoprecipitated protein.

Our goal was to manipulate the impact of LPS binding to TLR4 in vivo, and ultimately to impact the pathways involved in sickness behavior. Considering that LPS may be acting directly on TLR4 receptors in accessible regions of the CNS and in peripheral immune cells [2], [11] we established a technique that could target CNS TLR4 receptors. Accordingly, we developed interfering peptides coupled to a truncated Tat carrier sequence [15] in an attempt to block TLR4 signalling in brain slices, and subsequently examined their efficacy in preventing TLR4 activation *in vivo*. Specifically, the peptides were designed to block TLR4-MyD88 binding via the intracellular TIR domain induced by LPS activation of TLR4 (Figure 1A). We based our sequence on epta-peptides directed against the BB-loop within the TIR domains of TLR4 (Tat-MyD88) and MyD88 (Tat-TLR4) [16], [17].

We first determined whether these interfering peptides entered cells in brain slices after bath application *in vitro* or crossed the BBB and entered CNS cells after i.p. injections *in vivo*. Dansylated Tat-MyD88 was injected intraperitoneally (i.p.; 6 µg/kg) into mice and 30 minutes later acute brain slices were prepared for immediate examination using two-photon laser scanning microscopy (TPLSM). Strong dansyl fluorescence was detected within cells in the hippocampus, in contrast to vehicle injected controls (Figure 1B,C), indicating that the Tat-fused peptides could cross the BBB and permeate CNS cells. Similarly fluorescence was observed within cells in brain slices when brain slices were incubated in ACSF with dansylated Tat-MyD88. These observations of labelled Tat-MyD88 peptides in CNS cells show that these peptides can enter cells where they potentially have access to the intracellular binding site of MyD88 and TLR4 receptors.

We next tested whether Tat-MyD88 effectively blocked the interaction between TLR4 and MyD88 under conditions where we saw that the dansylated peptides entered cells. We tested their efficacy in the whole brain by assessing their ability to prevent protein-protein interactions via co-immunoprecipitation. Mice were injected (i.p. 6 µg/kg) with either vehicle (control), Tat-MyD88 peptide, or a scrambled version of the MyD88 sequence coupled to Tat (Tat-scram) and whole brain lysates were prepared 30 minutes later. Western blots of immunoprecipitated brain lysate prepared from mice injected with Tat-MyD88 showed a reduction in the intensity of the MyD88 band co-immunoprecipitated using anti-TLR4 antibody (62.03±2.73 a.u.) compared to untreated control (100.00±12.45 a.u., p = .041) and Tat-scram treated (103.94±6.67 a.u., p = .004; Figure 1D,E). Likewise, the reverse co-immunoprecipitation of TLR4 using the MyD88 antibody was also diminished in mice injected with Tat-MyD88 (50.63±7.53 a.u.) compared to untreated control (100.00±3.58 a.u., p = .004) and Tat-scram treated (98.92±3.84 a.u., p = .005). No change was observed in the co-immunoprecipitation of either MyD88 with TLR4 (p = .794), or TLR4 with MyD88 (p = .847) when Tat-scram treated animals were compared to untreated controls (Figure 1D,E). This data reveals that i.p. injections of the Tat-conjugated interfering peptide Tat-MyD88 are capable of blocking interactions between MyD88 and TLR4 in the brain *in vivo*.

**Figure 2:**
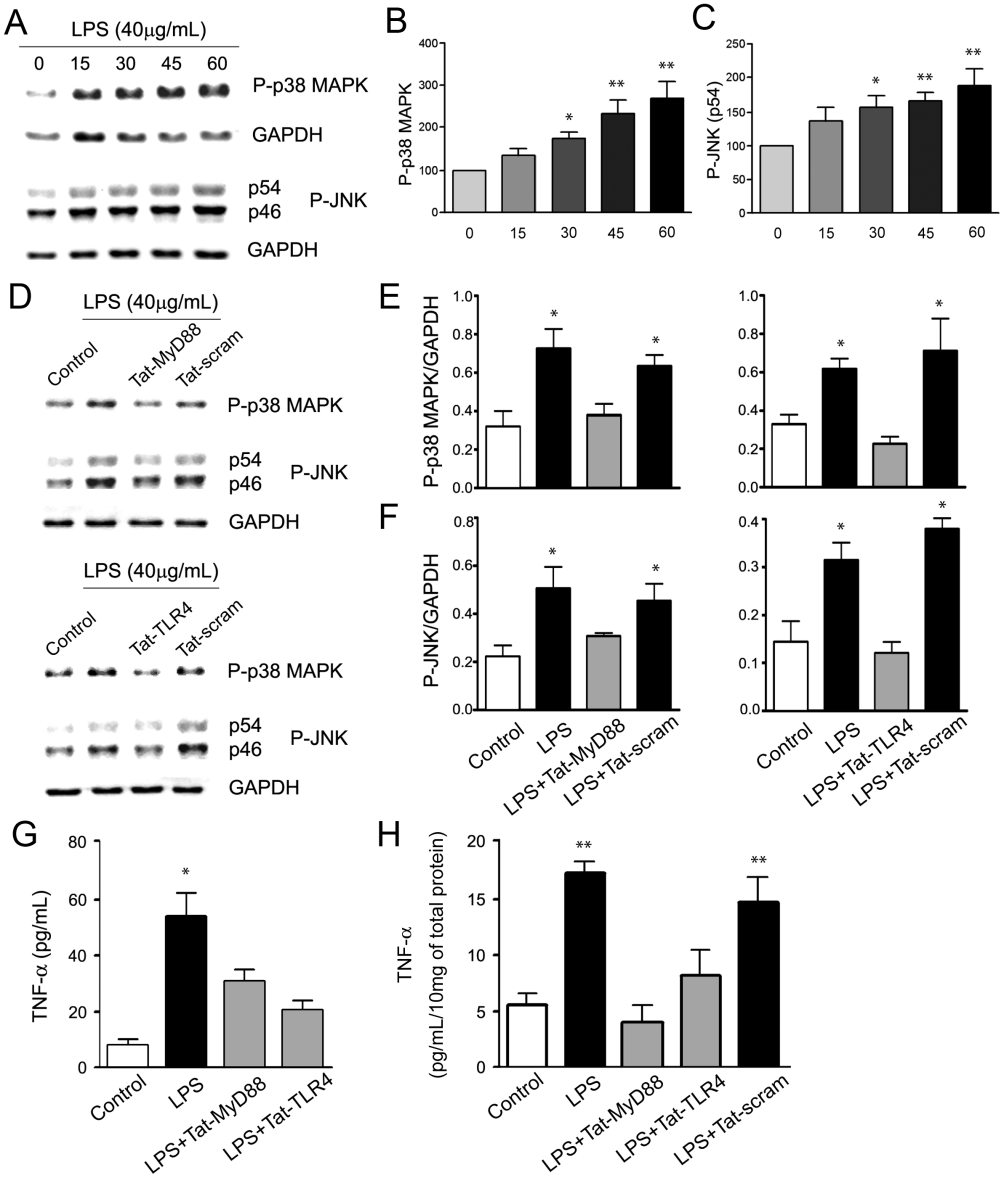
Time course of kinase activation and TNF-α formation following LPS treatment, and the inhibition by Tat-MyD88 and Tat-TLR4. A. Representative blots showing P-p38 MAPK and P-JNK rapidly increased in brain tissue following LPS treatment. GAPDH was monitored as a loading control. B,C. Quantification of the increased P-p38 MAPK and P-JNK levels over 60 minutes following LPS treatment. D-F. P-p38 MAP kinase and P-JNK increases from LPS were attenuated by Tat-MyD88 and Tat-TLR4. D. Representative blots of kinase activation following various treatments. E. Quantification of P-p38 MAPK normalized to GAPDH levels. F. Quantification of P-JNK normalized to GAPDH levels. G,H. LPS treatment increased TNF-α levels, and this increase was blocked by Tat-TLR4 and Tat-MyD88. Quantification of TNF-α levels using ELISA in acute brain slice (G) parallels results found in whole brain lysates of injected animals (H).

The disruption of co-immunoprecipitation supports the possibility that Tat-interfering peptides cause functional disruption, which we tested by examining the efficacy of Tat-MyD88 and Tat-TLR4 to inhibit LPS activation of second messenger pathways and cytokine production in both brain slices and *in vivo*. We began by determining the time course of downstream kinase activation in acutely prepared brain slices treated with LPS. Lysates were prepared 0, 15, 30, 45 or 60 minutes following treatment of slices with LPS (40 µg/mL; 45 min), and membranes were probed for phospho-p38 MAPK (P-p38MAPK), phospho-JNK (P-JNK), and GAPDH, which was used as a loading control (Figure 2A). Increases in both P-p38MAPK and P-JNK (normalized to GAPDH intensity) were significant at 30 (172.63±17.83 a.u., p = .003; 155.91±17.38 a.u., p = .004), 45 (235.65±29.42 a.u., p = .001; 166.10±13.32 a.u., p = .004), and 60 (271.14±38.85 a.u., p<.001; 189.94±23.41 a.u., p = .001) min following LPS treatment (Figure 2B,C). However pre-incubation (1 hour) of brain slices with either Tat-MyD88 (3 µM), Tat-TLR4 (3 µM) prevented the same LPS stimulation (40 µg/mL; 45 min) from increasing P-p38MAPK (p = .654; p = .395) and P-JNK (p = .386; p = .686) levels (Figure 2D-F). In contrast, matched brain slices pre-incubated with a scrambled sequence of either the Tat-MyD88 or Tat-TLR4 (0.635±0.057 a.u., p = .027; 0.710±0.169 a.u., p = .005) still showed the LPS-stimulated increases (Tat-scram; 3 µM). Therefore Tat-MyD88 and Tat-TLR4 peptides blocked the first steps involving second messengers that result from the functional activation of TLR4 receptors by LPS.

We next tested the efficacy of these peptides on the production of the cytokine TNF-α which is further downstream from the second messenger activation [18], in both brain slices and *in vivo*. Brain slices were pre-incubated with either vehicle, Tat-MyD88, Tat-TLR4 or Tat-scram I hr before a 2 hr LPS treatment (40 µg/mL). ELISA detection of TNF-α from brain slice supernatant showed using ANOVA that the LPS-induced (17.38±1.00 a.u., p = .001) cytokine production effect was blocked by Tat-MyD88 (p = .532) and Tat-TLR4 (p = .287) but not by Tat-scram (14.70±2.17 a.u., p = .003) (Figure 2G). After determining that these Tat interfering peptides were effective in brain slices we next investigated whether they could effectively prevent *in vivo* activation of TLR4 receptors by LPS. We tested their efficacy *in vivo* by i.p. injections (6 µg/kg) of Tat-scram, Tat-MyD88 or Tat-TLR4 I hr before i.p. injection of LPS (0.5 mg/kg), then measured TNF-α levels in whole brain lysate using ELISA after 45 min. Recent studies have demonstrated that i.p. injections of LPS increase cytokines in the brain by two principal mechanisms. LPS directly triggers cytokine release from microglia by diffusing into the brain at the circumventricular organs that lack a functional BBB and second by the peripheral actions of LPS triggering the release of cytokines that are then transported into the brain [2], [19]. In our experiments LPS injections triggered robust increases in TNF-α levels in control CNS tissue after 45 min (53.83±7.94 a.u., p<.001). In contrast, the increase in brain cytokines from LPS injections were significantly depressed by pre-treatment with either Tat-MyD88 (p = .030) or Tat-TLR4 (p<.001) but not by Tat-scram (Figure 2H). Thus, we were able to demonstrate the efficacy of blocking TLR4-MyD88 interactions by Tat-interfering peptides by monitoring the second messenger activation and the subsequent formation of the cytokine TNF-α.

**Figure 3:**
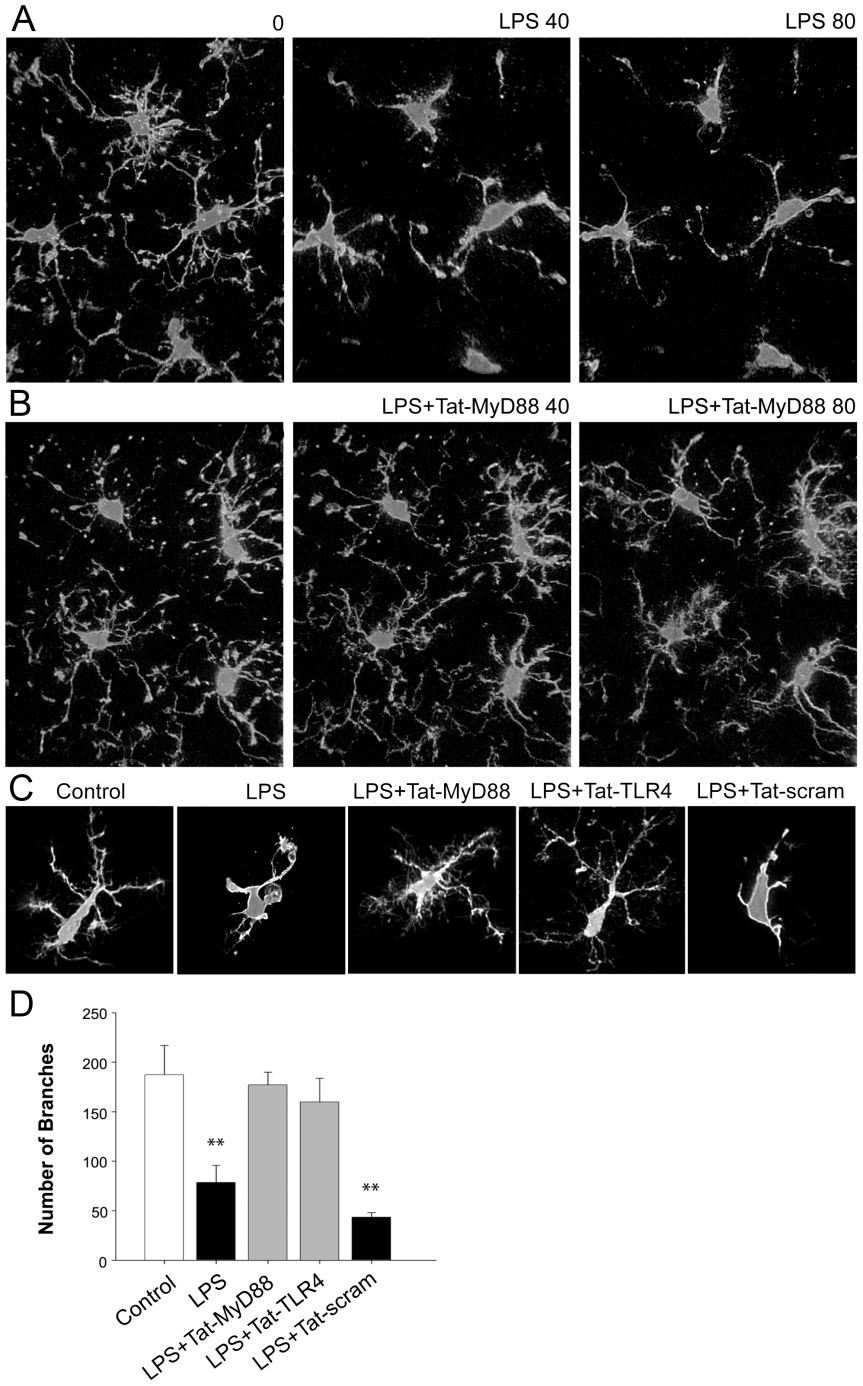
Time course of LPS-induced microglia morphology changes visualized using 2-photon imaging and the block by Tat-TLR4 and Tat-MyD88. A. Series of images at 0, 40 and 80 minutes following application of LPS showing the progression to amoeboid shape in microglia. B. Series of images at 0, 40 and 80 minutes showing Tat-MyD88 blocked the amoeboid shape change normally induced by LPS. C. Representative images of single microglia following individual treatments. D. Quantification of the number of branches of microglia following treatment.

The stimulation of TLR4 receptors in microglia has been shown to change their morphology from the resting ramified appearance to an amoeboid shaped [20]. We imaged dynamic changes in live EGFP positive microglia [21] prior to and during LPS application to determine the impact of blocking TLR4 signalling on the dramatic morphology changes normally induced in microglia by LPS. Microglia in the deeper healthy parts of brain slices were observed to have normal morphology using TPLSM, with ramified processes. Careful handling of acute slices ensured that only cells at the surface (<10 um) of the acute slice appeared to be affected by the slicing process, and in the depths at which we imaged neurons and astrocytes were healthy, and microglia did not appear activated. Under control conditions, microglia in acutely prepared brain slices exhibit the typical ramified morphology of resting microglia with numerous long branches, and multiple filopodia [22] (Figure 3A) similar to their appearance *in vivo* [23]. Staining of fixed tissue has shown that microglia *in vivo* acquire an amoeboid shape in response to brain injuries or to immunological stimuli such as LPS [24]. The morphological changes in microglia reflect profound functional changes in these cells because it is known that the release of cytokines and other signalling factors into the surrounding tissue [25] is enhanced when microglia acquire amoeboid morphology [24]. Using time-lapse TPLSM, we observed the progression of LPS-induced morphology changes in large fields of view where multiple microglia were visible (Movie S1). Within 10 min we observed the first indications that LPS application (t = 0) changed microglia morphology from the typical branched and ramified morphology (Figure 3A), and by 40 minutes, the majority of branches were lost and the cells were amoeboid (Movie S1). The amoeboid morphology of microglia persisted throughout the remainder of imaging (80 minutes). In comparison, when slices were pre-incubated with Tat-MyD88 or Tat-TLR4, the transition from ramified to amoeboid characterized by branch loss was not observed at either 40 or 80 minutes following LPS treatment (40 µg/mL; Figure 3B). The inhibitory effects of the Tat-interfering peptides on microglia morphology changes was quantified in a separate set of experiments by analysing the number of branches in three dimensional reconstructions using Imaris software of individual microglia (n = 21 cells per group). One way ANOVA demonstrated a significant main effect of treatment (F_4,104_  = 212.88, p = <.001). The number of branches in microglia were significantly reduced by LPS (Figure 3C, D; control = 187.5±29.5 branches, LPS = 78.5±17.5 branches; p = 0.011). This morphological transformation was blocked by pre-incubation with either Tat-MyD88 or Tat-TLR4, and was not significantly different from control (Figure 3C, D; p = 0.722 and p = 0.369 respectively). In contrast, microglia in brain slices pre-incubated with Tat-scram, showed a change in branch number induced by LPS that was similar to LPS treated slices (Figure 3D; Tat-scram = 43.5±4.5 branches; p = 0.04).

**Figure 4:**
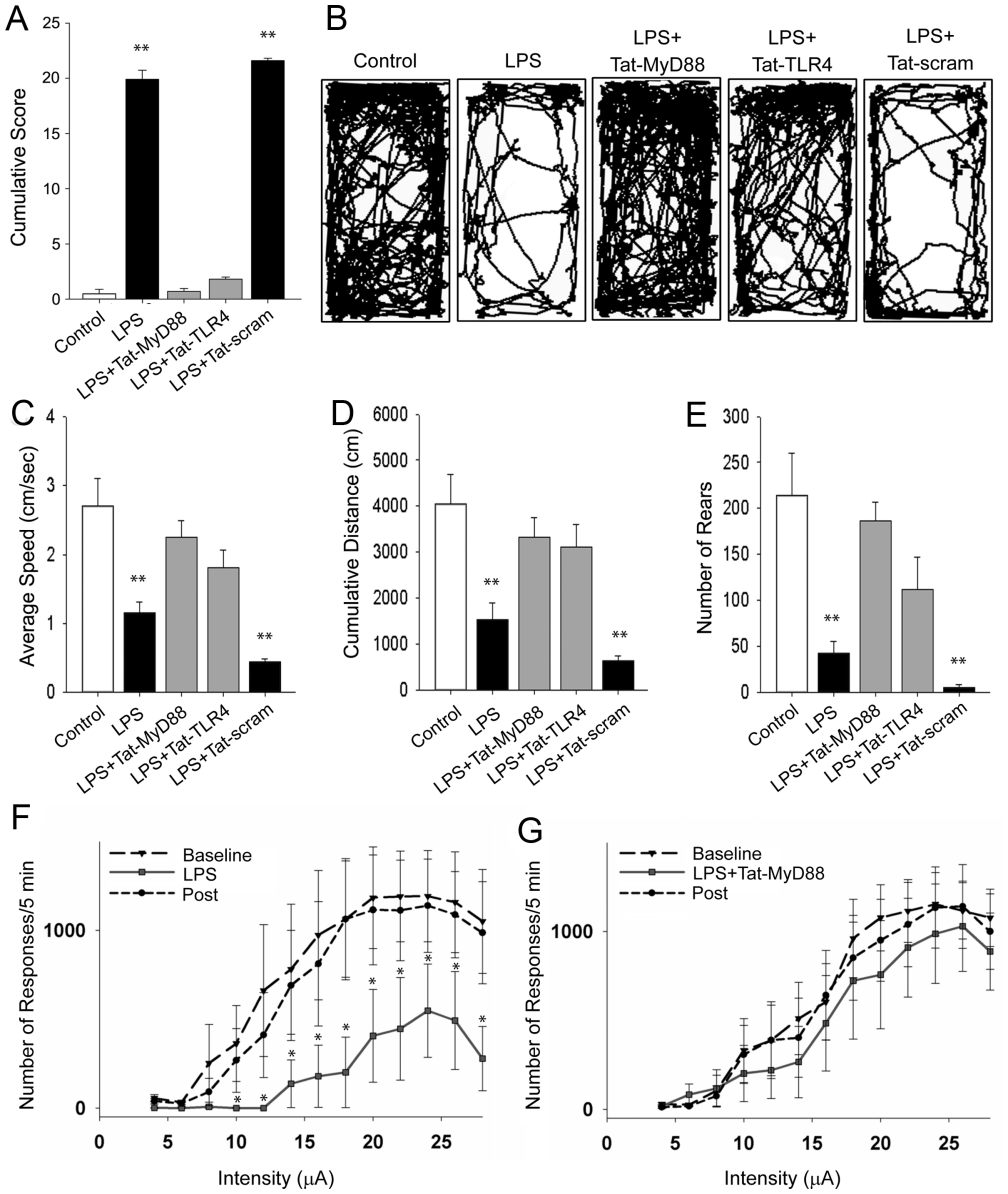
LPS induced sickness behavior was blocked by Tat-MyD88 and Tat-TLR4 as assessed in mice using a modified SHIRPA screen and in the novel home cage following various treatments. A. Cumulative score of sickness obtained using the SHIRPA screen. B. Representative paths of mice over 30 min in the home cage showing decreased exploration induced by LPS, and effective block by Tat-MyD88 and Tat-TLR4 but not Tat-scram. Average speed travelled (C), cumulative distance travelled (D), number of rears in the home cage (E). F, G. Assessment of sickness behavior in rats using intracranial self stimulation. F. Number of responses per minute during baseline, LPS treatment, and following LPS treatment. G. Number of responses per minute during baseline, LPS plus Tat-MyD88 treatment, and following LPS plus Tat-MyD88.

The ability of Tat-MyD88 and Tat-TLR4 to prevent many of the cellular actions of LPS such as second messenger stimulation, cytokine formation and transformations of microglia to amoeboid shapes encouraged us to test their effectiveness at treating LPS-induced sickness behavior. We began by assessing mice given LPS (0.5 mg/kg) or LPS (0.5 mg/kg) plus peptide treatments (6 µg/kg) on a number of basic behavioral indices of sickness including reflexive or motor and motivational or hedonic functions (Table S1). Mice were scored for the extent to which they displayed each of the 11 indices of sickness and a cumulative score was calculated (n = 10 mice per group). One way ANOVA demonstrated a significant main effect of treatment (F_5,59_  = 597.53, p = <.001). Control mice scored in the lowest category on each of the measures of sickness, with an average cumulative score of 0.50 (±0.40; Figure 4A). In contrast, mice assessed 30 minutes after LPS treatment scored high on each of the measures, with a cumulative score averaging 19.90 (±0.84) that differed significantly from control mice (p<.001). Similar results were observed for mice pre-treated with the Tat-scram peptide and LPS (21.67±0.24) compared to control mice (p<.001). When mice were pre-treated with either Tat-MyD88 or Tat-TLR4 peptides, we observed a remarkable prevention of LPS-induced sickness as reflected in the cumulative behavioral scores (Tat-MyD88:0.70±0.26; Tat-TLR4:1.80±0.20), which did not differ from control mice (p = .752 and p = .107).

To evaluate LPS-induced sickness behavior and the effectiveness of the Tat fused interfering peptides further, we observed mouse behavior in a novel home cage. Mice were i.p. injected with LPS (0.5 mg/kg) and returned ot their homecage for 30 min, with a subset treated with Tat-MyD88, Tat-TLR4 or Tat-scram (6 µg/kg) 30 min prior to LPS treatment. Mice were then singly placed into a novel home cage environment and allowed to explore freely for 30 min. Representative path plots from each of the groups tested illustrate striking differences among the groups (Figure 4B). Using Noldus Ethovision software for quantification, average speed (Figure 4C), cumulative distance traveled (Figure 4D), and total number of rears (Figure 4E) were used to provide objective measures of motor activity as an index of the severity of sickness. One way ANOVA demonstrated a significant main effect of treatment on open field performance, with speed (F_4,48_  = 12.07, p = <.001), distance (F_4,48_  = 8.43, p<.001), and rearing (F_4,48_  = 10.35, p<.001) as measures. Mice treated with LPS alone, or LPS plus pre-treatment with Tat-scram showed significant reductions in average speed (1.15±0.166, p<.001; 0.43±0.045, p<.001) and cumulative distance travelled (1531.44±369.10, p<.001; 691.75±113.97, p<.001), as well as the total number of rears (42.40±12.66, p<.001) compared to control (vehicle injected) mice. In contrast, mice pre-treated with either Tat-MyD88 or Tat-TLR4 prior to LPS treatment showed an increase in average speed (2.25±0.23, p = .003; 1.88±0.20, p = .038), total distance (3330.95±429.16, p = .008; 2808.68±206.98, p = .022), or number of rears (187.10±19.60, p = .001; 121.10±16.52, p = .005) in the novel home cage, compared to LPS treated animals. These differences in motor activity between control, LPS and interference peptide-treated groups are immediately observable when representative mice are viewed concurrently in the Movie S2.

Given the prevalence of decreased motivation in sickness behavior, we examined the effectiveness of the TLR4-MyD88 interfering peptides on the behavioral response of rewarding intracranial self-stimulation (ICSS). ICSS accesses self-motivated behaviors to acquire brain-stimulation reward related to activation of brain dopamine neurons [26], and consequently may be used to assess the direct effect of LPS on brain function in the absence of peripheral effects [27], [28]. Using the titrated protocol of this assay will allow us to examine the effects of sickness and peptide treatment on underlying motivational states. Two way repeated measures ANOVA revealed a significant interaction (intensity x treatment; F_24,272_  = 2.60, p<.001) between intensity (F_12,272_  = 11.11, p<.001) and treatment (F_2,272_  = 10.79, p = .002). In LPS treated animals (0.5 mg/kg), a significant reduction in the number of responses per 5 minutes (least square mean 207.40±92.03) was observed compared to baseline (least square mean 773.25±92.03; p = .003) and post-treatment (least square mean 674.79±92.03; p = .004) sessions (Figure 4F). In contrast, when animals were pretreated with Tat-MyD88 (6 µg/kg) prior to LPS (0.5 mg/kg), this reduction in the number of responses was not observed (Figure 4G). This result indicates that peptides interfering with TLR4-MyD88 can reduce the effects of sickness on motivation for self-stimulation. The behavioral data demonstrates that the peptides interfering with TLR4-MyD88 can effect both the motoric and hedonic effects of sickness behavior suggesting that peptide is capable of influencing multiple systems in the brain.

## Discussion

Taken together, our results show a remarkable ability of two different Tat-interfering peptides to prevent the downstream actions of TLR4 receptor stimulation at the molecular, cellular and behavioral levels. Although similar peptides have previously been developed, we have been the first to show a behavioral impact of blocking TLR4-MyD88 interaction, likely mediated by a rescue of microglia morphology changes and cytokine production that are normally induced by LPS. These peptides cross the BBB and enter cells where they disrupt the protein-protein interactions between TLR4 and MyD88. The peptides mimicked the key sequences necessary for dimerization and interaction of MyD88 and TLR4 TIR domains. Natural mutations of this key sequence in the TLR4 receptors were previously discovered to explain the unresponsiveness of a specific strain of rat to LPS [29]. We found that interfering peptides that mimic either the sequence of TLR4 receptors or the sequence on the recognition site on MyD88 prevented the co-immunoprecipitation of these proteins and the ability of LPS to activate second messengers and increase cytokine formation in intact tissue in brain slices and *in vivo*. Using two photon imaging we have further shown the dynamic morphological changes that microglia can undergo in responses to LPS and that these changes can be mediated by TLR4 signaling. In addition these Tat-interfering peptides were remarkably effective at preventing the behavioral syndrome that accompanies sickness caused by LPS. When mice were administered LPS by peripheral i.p. injection, a series of behavioral changes occurred within a one hour period, as previously reported [2]. Following treatment with either Tat-MyD88 or Tat-TLR4, but not a Tat-scrambled peptide, there was a complete absence of motoric (behavioral screen) and motivational (behavioral screen and titrated ICSS) effects of LPS-induced sickness which we attribute to the direct action of the peptide on brain function.

The sickness behavior of animals, triggered by the inflammatory release of cytokines, mirrors the well known symptoms of sickness in humans which include fatigue, loss of appetite and cognitive changes. It is also becoming evident that sickness and inflammation are important contributors to the occurrence of depressive episodes [2]. Therefore, these peptides represent a novel means of blocking the behavioral impact of sickness, and potentially an effective strategy for alleviating symptoms of depression induced by chronic inflammation and sickness. Our detailed description of a molecular target linking inflammation and sickness to motivational states provides valuable insight into pathways involved in the cross talk between the CNS and the immune system. In addition to acute sickness and depression, TLR4 activation in microglia has also been linked to neurodegeneration [30]. Therefore, Tat-interfering peptide strategies may also be useful for investigations of potential therapeutic interventions in CNS diseases in which activated microglia may be a causal factor in the underlying pathology. Ultimately, these findings allow for the further translation of our knowledge about the communication between the brain and the immune system and will create new therapeutics to increase the quality of life for sick patients.

## Materials and Methods

### Peptide Design

The generated peptides (Anaspec, San Jose, CA.) were based on epta peptides [17] with sequences mimicking the BB-loop of TLR4 and MyD88 TIR domains, preceded by a truncated Tat sequence (2). Sequences for the peptides are as follows: Tat-TLR4: YGRKKRRQRRR-RDFIPGV; Tat-MyD88: YGRKKRRQRRR-RDVLPGT; Tat-Scram: YGRKKRRQRRR-PTDLVRG

### LPS and Peptide Administration

LPS was dissolved in saline at a concentration of 40 µg/mL and either injected intraperitoneally for *in vivo* experiments, or bath applied for experiments conducted in slice. Both the Tat-TLR4 and Tat-MyD88 peptides were dissolved in saline to a concentration of 20 µg/mL, and again were either bath applied or injected depending upon the experiment. In experiments where both LPS and peptide treatments were used, peptide injection preceded LPS by 30 min.

### Slice preparation and solutions

Brain slices were obtained as described previously [31] from CX3CR1-EGFP [21] transgenic mice aged 21–40 days postnatal. Slices were stored at room temperature (20 to 23°C) for 1 hr before imaging in an oxygenated artificial cerebrospinal fluid (aCSF) containing (in mM): NaCl 126, KCl 2.5 or 4.2, NaHCO_3_ 26, glucose 10, MgCl_2_ 2, NaH_2_PO_4_ 1.25 and CaCl_2_ 2. Slices were transferred to a recording chamber and perfused with oxygenated aCSF at a rate of 1–3 mL/min and maintained at either 25°C with an inline heater (Warner Instruments).

### Two-photon imaging

We performed imaging with a two-photon laser-scanning microscope (Zeiss LSM510-Axioskop-2 fitted with a 40X-W/0.80 numerical aperture objective lens) directly coupled to a 10 W Chameleon ultrafast laser. EGFP was typically excited at 820 nm and epifluorescence was detected with external detectors. For acquiring images laser intensities were <25 mW at the tissue and there was no photobleaching nor was there any evidence of cellular damage during extensive scanning to obtain time lapse images. The laser intensity was carefully monitored in all instances and kept comparable between all experiments. Imaging was done at depths in brain slices >50 µm and up to 100 um. The mean depth for imaging lesions was 75 um. Z-stacks were taken in 0.5 µm steps and covered a field of 64.5 µm×64.5 µm. The mean scan time for z-stack was approximately 1 min and 18 sec.

3D reconstruction of microglia, and automated assessment of the number of branches was performed using Imaris 5.0 (Bitplane AG, Zurich, Switzerland). The Gaussian filter was set to 0.5 uM in accordance to the dimensions of the PSF of the microscope.

### Western Blotting and Co-Immunoprecipitation

All samples were boiled in SDS page sample buffer with DTT for 10 min. After SDS page and transfer, nitrocellulose membranes were probed with anti-TLR4 and anti-MyD88 followed by alexa 680 and IRD800 conjugated secondary antibodies for detection using an Odyssey machine (Li-Cor), or HRP conjugated secondaries for ECL detection.

For co-immunoprecipitation, brain tissue from either Tat-TLR4 injected, Tat-scram injected, or untreated mice was rapidly harvested and homogenized in TEEN buffer (50 mM Tris-HCL, 1 mM EGTA, 150 mM NaCl) plus 0.2%SDS and 0.8% TWEEN, supplemented with PMSF, and protease inhibitor tablet (1 for 10 mL; Roche Applied Science). Following a 30 min lysis, samples were spun at 14000 rpm for 10 min at 4°C, and the supernatant was transferred to a clean tube. Lysed protein was incubated with either anti-TLR4 (Cedarlane) or anti-MyD88 (Cedarlane) for 1 hr at 4°C. Sepharose beads (company) were washed 3× in TEEN buffer and added to tubes containing lysed protein-antibody, and incubated at 4°C for 1 hr. Following incubations, beads were washed 3× in the above described TEEN buffer, and subjected to SDS-page described above.

### ELISA

Enzyme-linked immunosorbent assays (ELISA) were performed according to manufacturer instructions (R & D systems, Minneapolis, MN). In brief, hippocampal brain slices were incubated and treated in a homemade chamber (using 12 multi-well plates) equipped with continuous aeration with 95% O_2_/5% CO_2_. Slices were treated with LPS (40 µg/ml) in the absence or presence of Tat peptides. The latter were pretreated for 30 min prior to LPS application. The cell-free supernatants were used for analysis of TNF-α production. For in vivo experiments, mice were i.p. injected with LPS (0.5 mg/kg) and hippocampi were surgically removed for protein assay and mouse TNF-α ELISA (eBioscience, San Diego, CA). Tat peptides (6 µg/kg of body weight) were intraperitoneally injected 30 min prior to LPS administration.

### Cumulative Behavioral Score

The behavioral screen were based on the modified SHIRPA protocol employed by European Mouse Phenotyping Resource of Standardised Screens (EMPReSS) designed to evaluate phenotypes of mouse strains [32], [33].

The behavioral screen were based on the modified SHIRPA protocol employed by European Mouse Phenotyping Resource of Standardised Screens (EMPReSS) designed to evaluate phenotypes of mouse strains (Brown et al., 2005, Brown et al., 2006). Details of the assessments used and the scoring are as follows:

-Body position
  Normal: sitting or standing
  1 =  Occasional hunching of back (kyphosis)
  2 =  Intermittent but pronounced kyphosis
  3 =  Lying on side, or prone
-Basal activity (square crossings)
  Normal: vigorous scratching, grooming, moderate movement
  1 =  Vigorous, rapid/darting movement
  2 =  Repeated rearing on hind legs and/or repeated vertical leaping
  -Palpebral Closure
  Normal: eyes open
  1 =  Eyes 1/2 closed
  2 =  Eyes closed
-Piloerection
  1. 0 (Normal): None, coat smooth
  2. 1 =  Coat stands on end
-Transfer arousal
  0 (Normal): brief freeze followed by active movement
  1 =  Prolonged freeze, then slight movement
  2 =  No freeze, immediate movement
  3 =  Extremely excited ("manic")
-Tail position during forward motion
  1. 0 (Normal): horizontally extended tail during locomotion
  2. 1 =  Dragging
  3. 2 =  Elevated/Straub Tail
-Touch escape from finger stroke on back from above
  1. Normal: moderate (rapid response to light stroke)
  2. 1 =  Vigorous (escape response to approach)
  3. 2 =  No response
-Pinna reflex (to touch of the proximal part of the inner canthus)
  1. Normal: active retraction, moderately brisk flick
  2. 1 =  Hyperactive, repetitive flick
  3. 2 =  None
-Corneal reflex (to light touch of the cornea)
  1. Normal: active single eye blink
  2. 1 =  Multiple eye blink
  3. 2 =  None
-Body tone (resistance to finger press on body cavity)
  1. Normal: resistance
  2. 1 =  Flaccid, no return of cavity to normal
  3. 2 =  Extreme resistance, board like
-Limb tone (limb resistance to gentle finger tip pressure on hind paw)
  1. Normal: resistance
  2. 1 =  Extreme resistance
  3. 2 =  No resistance

### Novel Home Cage Behavior

The behavior of mice in the novel home cage was assessed using the Noldus Ethovision automated Video Tracking system (Noldus, Wageningen, The Netherlands). Parameters including total distance travelled, average speed, and number of rears were assessed. Results from tracking analysis were analyzed using ANOVA to compare means.

### Intra-cranial Self Stimulation

Surgery: Rats were anaesthetized with xylazine (7 mg/kg i.p.) and ketamine hydrochloride (100 mg/kg i.p.), and placed in a standard stereotaxic apparatus. The dorsal surface of the skull was exposed and a single hole was drilled to allow implantation with a stainless-steel bipolar electrode. Electrodes were directed at a site in the medial forebrain bundle corresponding to the level of the posterior lateral hypothalamus (AP, ×0.5 mm from bregma; ML, +1.7 mm; DV, x8.3 mm from dura; tooth bar, 5.0 mm above the interaural line). Electrodes were secured to the skull with surgical stainless-steel screws and dental acrylic cement. All animals were allowed to recover from surgery for at least 1 wk before starting ICSS training.

ICSS Training: Training and testing were conducted in 8 Plexiglas boxes (30r30r24 cm), housed within sound-attenuating chambers. Depression of a lever delivered a sinewave current (60 Hz) of fixed duration (200 ms), via a flexible lead connected to the chronically implanted intracranial electrode assembly. During the initial training period, the current was set at 16 mA, and only those animals that maintained consistent lever pressing were used for the second stage of training. This stage consisted of training subjects on an ascending series rate-intensity protocol, whereby current intensities were preset by a computer (Nova-3; Manx software) and increased in 2 mA steps, from an initial value of 8 to 28 mA. Five priming pulses of stimulation were delivered to each animal at the beginning of the first minute of testing at a given current level. The number of bar presses was recorded for the subsequent 4-min period, after which the current intensity was set at the next level. Data collection was controlled by the computer and individual rate–intensity curves were plotted daily for each subject, from which three measures were calculated; the current at which responding was half maximal (M50), the minimum current required to maintain a threshold level of responding of 30 presses per minute, and the asymptotic level of responding.

After stable levels of ICSS responding had been achieved (M50<¡10%, for 3 consecutive days), animals were rank ordered based upon their level of responding over the previous three baseline sessions. Rats were then assigned sequentially to 4 groups, from the highest to the lowest M50 values. Two groups (n = 7, 8 per group) were assigned to receive administration of LPS (0.5 mg/kg), while two groups (n = 7 per group) received vehicle.

Electrode placements were verified at the conclusion of the experiment by sectioning 50 mm coronal slices of the brain at the level of the lateral hypothalamus. Brain sections were stained with Cresyl Violet and the locations of the electrode tips were recorded.

### Statistical Analysis

Data were expressed as mean ± s.e.m. and the statistical significance of differences in mean values was assessed by t-test, one- or two- wayanalysis of variance (ANOVA), or two-way repeated measures ANOVA; Student Newman Keuls post hoc comparison was used as appropriate. Differences among means were considered significant at values of *: p≤.05, **: p≤.01, ***: p≤.001.

## Supporting Information

Table S1 **Table outlining behavioural assessments used and whether they inform us about movement and motility (motoric) and/or the brain reward and motivational (hedonic) systems.** Check marks indicate that the behaviour contains elements of these parameters.
(DOCX)

Movie S1 **Live 2-photon imaging of the effects of LPS on microglia morphology, and blockade of LPS-induced microglia morphology changes by treatment with Tat-MyD88.** Under control conditions, microglia in acutely prepared brain slices exhibit the typical ramified morphology characterized by numerous long branches, and multiple filopodia. Within 10 min following LPS treatment, we observed the first indications of morphology change, and by 40 minutes, the majority of branches were lost and the cells were amoeboid. The amoeboid morphology of microglia persisted throughout the remainder of imaging (80 min). In comparison, when slices were preincubated with Tat-MyD88, the transition from ramified to amoeboid characterized by branch loss was not observed at either 40 or 80 minutes following LPS treatment.
(MOV)

Movie S2 **Simultaneous viewing of an untreated control mouse (blue), and mice treated with either LPS alone (green), or LPS plus Tat-MyD88 (red) demonstrates the striking differences in behavior.** Mice treated with LPS show very little exploratory behavior, take on a hunched posture, and show pronounced piloerection. In contrast, mice treated with LPS plus Tat-MyD88 show a smooth coat, normal posture, and high levels of exploratory behavior, comparable to untreated control animals.
(MOV)
