# Supplementary material for: Prevention of LPS-Induced Microglia Activation, Cytokine Production and Sickness Behavior with TLR4 Receptor Interfering Peptides
Source: PLoS One. 2013 Mar 28;8(3):e60388. doi: 10.1371/journal.pone.0060388 (PMC3610686; doi:10.1371/journal.pone.0060388)
Supplement: Table S1 — Table outlining behavioural assessments used and whether they inform us about movement and motility (motoric) and/or the brain reward and motivational (hedonic) systems. Check marks indicate that the behaviour contains elements of these parameters. (DOCX) [file pone.0060388.s001.docx]

Table S1.

| **BEHAVIOR** | **Motoric** | **Hedonic** |
| --- | --- | --- |
| Body Postion |  | ✓ |
| Basal activity (square crossings) | ✓ | ✓ |
| Palpebral Closure |  | ✓ |
| Piloerection |  | ✓ |
| Transfer arousal |  | ✓ |
| Tail position during forward motion | ✓ |  |
| Touch escape from finger stroke on back from above |  | ✓ |
| Pinna reflex (to touch of proximal inner canthus) | ✓ |  |
| Corneal reflex (to light touch of the cornea) | ✓ |  |
| Body tone (resistance to finger press on body cavity) | ✓ | ✓ |
| Limb tone (hindlimb resistance to gentle finger tip pressure) | ✓ | ✓ |
